# Passion and Persistence: Investigating the Relationship Between Adverse Childhood Experiences and Grit in College Students in China

**DOI:** 10.3389/fpsyg.2021.642956

**Published:** 2021-02-22

**Authors:** Shannon Cheung, Chien-Chung Huang, Congcong Zhang

**Affiliations:** ^1^School of Social Work, Rutgers University, New Brunswick, NJ, United States; ^2^Department of Youth Work Research, China Youth University of Political Studies, Beijing, China

**Keywords:** grit, adverse childhood experience, emerging adults, China, college students

## Abstract

Adverse childhood experiences (ACEs) are known to have deleterious effects on individuals across the life span, but less is known about how they affect grit, a strong predictor of achievements and well-being. This study seeks to investigate the effect of ACEs on grit in a sample of Chinese college students during the COVID-19 pandemic. Data were collected from 1,871 students across 12 universities in China. Findings indicated a significant effect of ACEs on grit, particularly abuse and neglect dimensions of ACE. Since grit is particularly important for professional success, those who have experienced abuse and neglect victimization may struggle throughout their education, and subsequently, in their careers. This calls for interventions to buffer the effects of ACEs on grit.

## Introduction

Studies have shown that, regardless of geographic location, almost two-thirds of youth have experienced a significant adverse event (see Carlson et al., 2019 for review). Adverse childhood experiences (ACEs), including abuse (psychological, physical, or sexual), neglect, household challenges such as violence perpetrated against mother and cohabitation with individuals who use substances or have mental illness or incarceration history [Felitti et al., 1998; Center for Disease Control and Prevention (CDC), 2020], from the first 18 years of life have been linked to a plethora of social and health issues in later childhood (Elmore et al., 2020), adolescence (Isohookana et al., 2013, 2016; Crandall et al., 2020; Zhang et al., 2020), and adulthood (Felitti et al., 1998; Brown et al., 2010; Gilbert et al., 2015; Schütze et al., 2020). For example, using data from a sample of children and adolescents ages 8 to 17, Elmore et al. (2020) found that youths with four or more ACEs had 2.29 times greater odds of experiencing depression than those with less than four ACEs. ACEs have also been found to be positively associated with increased risk of self-injury and suicide attempts among adolescents (Isohookana et al., 2013) and suicide attempts among adults (Dube et al., 2001; Fuller-Thomson et al., 2016). In fact, Dube et al. (2001) found that in a sample of over 17,000 adults, individuals with at least one ACE of any category had a two- to five-fold increased risk of attempted suicide. Research has found that the co-occurrence of ACEs is a major risk factor for health conditions ranging from substance use disorders and suicidality to chronic lung disease and cardiovascular disease (see Hughes et al., 2017 for review). Notably, the ACE paradigm has primarily examined the effects of ACEs through an additive lens, instead of focusing on any single form of maltreatment or abuse (Schütze et al., 2020).

The rise of literature on the strong relation between ACEs and an alarming variety of negative life outcomes is paralleled by the increasing prevalence of scholarship on constructs such as grit. Grit is defined as “perseverance and passion for long-term goals” (Duckworth et al., 2007, p. 1087). Grit describes one's ability to commit to an endeavor, regardless of the failures, setbacks, and adversities experienced along the way (Duckworth et al., 2007; Duckworth, 2016), a trait that is generally used to describe the capacity to adapt to and persevere through hardship (Duckworth, 2016; Stoffel and Cain, 2018).

Grit has been found to be important to bolstering academic achievement (e.g., Duckworth et al., 2007; Datu et al., 2018a; Alhadabi and Karpsinski, 2020) and professional success (e.g., Dam et al., 2019; Gruenberg et al., 2019; Musso et al., 2019; Meyer et al., 2020); beyond this, however, grit has also been linked to several indices of well-being in cross-cultural studies (Singh and Jha, 2008; Kleiman et al., 2013; Akbaǧ and Ümmet, 2017; Datu et al., 2018a). These indices include self-rated subjective well-being (Akbaǧ and Ümmet, 2017), happiness (Singh and Jha, 2008), and life satisfaction (Singh and Jha, 2008; Jin and Kim, 2017). Jin and Kim (2017) found that grit increases life satisfaction via increased sense of autonomy and competence, two constructs that may support emerging adults as they make significant strides in their transition to young adulthood (Arnett, 2007). While ACEs may positively predict poor health and life outcomes, these findings underscore the potential of grit to promote positive well-being. In fact, other studies have found that grit negatively predicts psychological distress (Datu et al., 2018a), substance use (Guerrero et al., 2016), and depression and anxiety among university students (Musumari et al., 2018), as well as buffer suicidal ideation risk (Kleiman et al., 2013), all outcomes that are disproportionately higher in populations with greater ACE scores (Dube et al., 2001; Isohookana et al., 2013; Fuller-Thomson et al., 2016; Carlson et al., 2019).

As the sample of interest lives in a cultural context that is distinct from that of Western countries, we note that Datu et al. (2016) found that in collectivist cultures, such as that of the Philippines, grit may be better conceptualized as a construct with two distinct components—*perseverance of effort* and *consistency of interests*. In fact, perseverance of effort (striving harder to accomplish goals despite challenges and hardships) specifically was found to significantly predict academic performance and subjective well-being, while consistency of interests did not (showing steady long-term interest) (Datu et al., 2016). These results differ from previous results based on data from American samples (Duckworth et al., 2007); it is possible that in collectivistic cultures, perseverance of effort is more relevant than consistency of interests. Further study on this construct, originally devised by Western scholars, in cross-cultural contexts is needed.

Though Duckworth et al. (2007) originally developed hypotheses about grit to explain academic and professional success, it has come to the attention of scholars that grit makes a difference outside of classrooms and offices and contributes to adolescents' and emerging adults' psychological well-being. More than this, grit has appeared to have some protective effect against poor mental health outcomes, to which those with ACE histories are prone (Dube et al., 2001; Isohookana et al., 2013; Fuller-Thomson et al., 2016; Carlson et al., 2019).

Thus far, literature has examined grit as a predictor of some outcome variable of interest; less is known about the antecedents of grit. In other words, there is a dearth of knowledge on what accounts for the variance in grit among individuals. Some studies have found that socioeconomic characteristics of individuals and their parents play a role in grit (Guerrero et al., 2016; Datu, 2017). Datu (2017) found that students' sense of relatedness with parents positively predicted students' grit. Meanwhile, parental employment and authoritative parenting style were positively associated with grit among Latino adolescents (Guerrero et al., 2016). Studies have also found that individual traits, such as goal commitment (Tang et al., 2019), self-efficacy (Guerrero et al., 2016), and mindfulness (Raphiphatthana et al., 2018) also positively predict grit.

Given that ACE literature has provided overwhelming evidence of the link between ACEs and negative psychological well-being and of the importance of grit in life outcomes, it is vital to understand the effects of ACEs on grit. This can shed light on policy and practice interventions. Importantly, ACEs have been shown to affects a multitude of factors important to the health, well-being, and achievement outcomes of adolescents, emerging adults, and adults. Less is known, however, about whether ACEs affect grit specifically. Understanding the relation between ACEs and grit may offer the first step in theorizing how ACEs negatively affect the life outcomes of adults who experience traumatizing events during their early life stages. The theoretical framework of this study is based on the trauma theory (Herman, 1992). Trauma theory posits that traumatic experiences, including those listed in the ACEs questionnaire, can impede the psychological well-being of individuals through the development of three symptom clusters: hyperarousal, constriction, and intrusion. Hyperarousal, a key symptom of posttraumatic stress disorder (PTSD), occurs when an individual's sympathetic nervous system is activated by a traumatic memory. The chronicity of hyperarousal reproduces a prolonged state of self-protective vigilance that is difficult to “turn off” or regulate. Often, in response, traumatized individuals experience another symptom cluster, constriction, wherein they may become physiologically, emotionally, and cognitively unresponsive to stimuli. While constriction can functionally help individuals avoid painful trauma-related responses, intrusion may break through, forcing the survivor to relive the trauma through fragmentary images and vivid sensations of the original experience, notably, in the form of nightmares (Herman, 1992). As a result, traumatic experiences overwhelm the victims, disrupt their inner schemas about safety and trust, and strip them of control, connection, and meaning in life. Empirical studies have provided evidence that ACEs are traumatizing events that have extensive harmful and long-term consequences on mental health such as increasing likelihood of depression (McLaughlin et al., 2012; Weder et al., 2014; Merrick et al., 2017), on physical health and risky behaviors such as substance use and delinquency (Herrenkohl et al., 2013; Huang et al., 2015; Mandavia et al., 2016), as well as on life satisfaction and well-being (McElroy and Hevey, 2014; Mosley-Johnson et al., 2019). Echoing Herman's (1992) conjecture that trauma may leave victims without any connection or meaning in life, increased mental health problems, such as depression, may lead to diminished passion in life. Similarly, escalated risky behaviors such as substance use and delinquency evidently reduce the perseverance to achieve goals and ambitions. As passion and perseverance are two key characteristics of grit, ACEs are likely to be negatively associated with grit.

To the authors' knowledge, grit has not yet been investigated in the context of ACEs, though the two have been demarcated as distinctive constructs. Similarly, although researchers are currently examining grit in college students (Bono et al., 2020) and healthcare professionals (Huffman et al., 2020) during the COVID-19 pandemic, grit among students residing in China during this time is understudied. The novel 2019 coronavirus disease spread rapidly throughout China and the rest of the world, posing a major threat to global public health (Ali et al., 2020). Given the ongoing pandemic, we sought to examine how COVID-19 may have affected college students' grit as well. Scholars have framed the global phenomenon of COVID-19 as a collective trauma, one that may lead to a rise in symptoms of prolonged grief disorder (PGD) (Kokou-Kpolou et al., 2020) and greater levels of stress, depression, anxiety, and PTSD, primarily in Chinese samples (Cao et al., 2020; Qiu et al., 2020; Wang et al., 2020). We do not know, however, how COVID-19, conceptualized as a potential, prolonged traumatizing event, may have affected grit within Chinese samples, particularly college students. Grit among college students may be especially salient given the context of the emerging adulthood life stage, which is characterized as a crucial transitory stage filled with significant increases in responsibility and independence (Arnett, 2007). This life stage is also “the peak age period” during which many societally discouraged behaviors take place, including risky behaviors like illegal drug use, binge drinking, and risky sexual behaviors (Arnett, 2007). Reduced grit at this life stage—in other words, lower perseverance and passion for long-term goals—may be correlated with the uptake of such behaviors, especially in the midst of a global pandemic. This concern provides rationale to assess ACEs and grit within a sample of college students. Guided by trauma theory (Herman, 1992), we hypothesized that ACEs and proximity to COVID-19 would be negatively associated with grit in Chinese college students.

## Methods

### Data and Sample

The data used for the present study came from the results of an online anonymous survey. Junior and senior students from 12 geographically diverse universities in China. We selected 12 universities from the north, south, east, west, and middle regions of China to ensure a diverse sample. Afterwards, we reached out to each university's department of social science, and in September 2020, we invited 2,229 junior and senior students to participate in the online survey. Reminders regarding survey participation were sent out 3 and 7 days after the initial invitation. By early October 2020, we had received responses from 1,881 students. After omitting 10 cases due to incomplete answers, we had final analytic sample of 1,871 college students. The response rate was 80%. This research protocol, including an informed consent process, was approved by the research review committee at one of the co-authors' university in China. Students were informed that participation was voluntary and that they could choose to discontinue the survey at any time.

Table 1 presents characteristics of sampled students. About two-thirds of the sample was female, mirroring the social science student population in China. The mean age of the sample was 20.62 and the majority of the sample was Han ethnicity (89.36%). Over half of the students (52.37%) had city household registration (HR), followed by 38.70% with rural HR, and 8.93% with city but prior rural HR. Majority of students reported that their parents were married (89.04%), while a small portion of students reported that their parents were divorced (6.89%). Most students (39.82%) reported their parents' highest education was college and above, followed by junior high school (28.11%), high school (25.17%), and elementary school (6.9%). The average family income was 90,990 RMB (about 13,580 USD) in the past year, with a standard deviation of 122,030 RMB (18,170 USD). About 25% of students reported that their families received at least one form of social welfare, such as low-income assistance, food subsidies, and other subsidies, in the past year. The average number of family members was 3.87. Finally, regarding the college composition, no college occupied the final sample more than 12%, ranged from 2.5 to 11.5%, reflecting the size of students in their social science departments.

**Table 1:** Descriptive statistics of sampled students.

	**Mean (S.D.)**
Sex [%]	
Female	66.97
Male	33.03
Age	20.62 (0.96)
Household Registration [%]	
Rural	38.70
City, rural before	8.93
City	52.37
Grade [%]	
Junior	60.72
Senior	39.28
Ethnicity [%]	
Han	89.36
Others	10.64
Parent Marital Status [%]	
Married	89.04
Separated	0.80
Divorced	6.89
Widowed	2.35
Others	0.91
Parent Highest Education Achievement [%]	
Elementary School and Below	6.90
Junior High School	28.11
High School	25.17
College and above	39.82
Family Income	90990 (122030)
Welfare Status	
No	74.72
Yes	25.28
Number of Family Members	3.87 (1.16)
College [%]	
College 1	7.11
College 2	9.57
College 3	6.25
College 4	10.85
College 5	10.15
College 6	7.06
College 7	6.41
College 8	11.54
College 9	11.12
College 10	2.46
College 11	6.89
College 12	10.58

*N = 1,871*.

### Measures

#### Grit

*Grit* was measured by the Short Grit Scale (Grit-S) developed by Duckworth and Quinn (2009). The 8 item-scale asks about intrapersonal competencies and assesses the extent to which individuals can maintain focus, interest, and perseverance when obtaining long-term goals. The Grit-S has shown with good psychometric properties, including high criteria and construct validity, as well as high internal consistency and test-retest reliability, in Chinese population (Li et al., 2018; Zhong et al., 2018; Luo et al., 2020). There are two subscales of grit, *perseverance of effort* and *consistency of interests* (referred to as *perseverance* and *consistency* hereafter), each containing 4 items. Example items for Grit-S include “Setbacks don't discourage me. I don't give up easily;” and “I have been obsessed with a certain idea or project for a short time but later lost interest.” Answers for each item ranged from 1 (Very much like me) to 5 (Not like me at all). We recoded the scores of each item so that higher scores indicated higher levels of grit. We calculate the average score of grit, as well as perseverance and consistency, by averaging the item scores. The range of the scores to each item ranged from 1 to 5. The Cronbach's alphas were 0.72, 0.83, and 0.69 for the full scale, the perseverance subscale, and the consistency subscale, respectively.

#### ACEs

The key independent variable, *adverse childhood experiences*, was measured by the Adverse Childhood Experience scale (ACE) and assessed ACEs during the respondent's first 18 years of life [Center for Disease Control and Prevention (CDC), 2020]. Ten items were used to measure ACE across three dimensions: abuse (3 items), neglect (2 items), and household challenges (5 items). The Cronbach's alpha was 0.69 for the above 10 items in this study. Example questions included “Did a parent or other adult in the household often: swear at you, insult you, put you down, or humiliate you?”, “Did you often feel that: No one in your family loved you or thought you were important or special?”, and “Did you live with anyone who was a problem drinker or alcoholic or who used street drugs?” Each affirmative answer was assigned one point. The sum of all affirmative answers represents the ACE score. A higher score indicates a higher frequency of experiencing adverse events in the first 18 years of life. In addition, we calculate the scores of three dimensions in ACE scale, as well as the percentages of each and the total ACE events.

#### Proximity to COVID-19

Proximity to COVID-19 infection was measured by asking students whether their family members or friends had tested positive or died due to COVID-19.

#### Covariates

This study included socioeconomic characteristics of the respondents as covariates. We collected information about respondents' age, sex (0 = male; 1 = female), ethnicity (0 = other, 1 = Han), and household registration (rural; city with prior rural registration; city). We also collected information about their parents' and family's background, including parents' marital status (married, separated, divorced, and widowed), parents' highest educational background (elementary school or below, middle school, high school, and some college or above), number of family members, annual family income in the last year, and welfare status (0 = no; 1 = yes) in the last year. In addition, since we sampled students from 12 colleges across China, we consider that different college characteristics may affect the grit of students. Thus, we took college into account by controlling for college as one of the covariates. Specifically, we used college-fixed dummies that were taken to be constant across individual colleges as covariates.

### Analyses

Descriptive analysis was performed to examine the distribution of each main variable. We then conducted regression analysis to estimate the association between key independent variables and the dependent variable while controlling for students' socioeconomic characteristics. The framework underlying this study posits that the extent of grit in college students is determined by ACEs, COVID-19 infection in family and friends, socioeconomic characteristics of the students, and college-level characteristics. The specification of the analytic model is represented by the following equation:

$$\begin{array}{l} Y_{\text{i}}=\alpha_{\text{i}}+\beta_{1}*\chi_{\text{i}}+C_{\text{i}}+\varepsilon_{\text{i}}, \end{array}$$

where *Y*i is grit of the subject i; α_i_ is the individual constant; χ is a vector of ACEs, COVID-19 infection in family and friends, and socioeconomic characteristics of subject i; C_i_ is the college for subject i, or college-fixed effect; β is a vector of regression coefficients; and ε_i_ is the cross-section error component. Note that with college-fixed effect, the model controls for differences across colleges. Ordinary least squares (OLS) regression was used for the analyses. All analyses were conducted using STATA software 16.0.

## Results

### Descriptive Statistics

Table 2 presents the descriptive statistics of grit, ACE, and COVID-19 infection. The sample had an average grit score of 3.07. Scores ranged 1 to 5 and had a standard deviation of 0.44. The average scores of perseverance and consistency were 3.28 and 2.86, respectively. More than one third (35.16%) of students reported that they experienced at least one type of ACE and 8% of them had at least three types of ACEs in childhood. ACE scores in the sample ranged 1–10 with a mean of 0.69 (SD = 1.28). In our study, average ACE subscale scores were 0.28 (SD = 0.63) for abuse, 0.15 (SD = 0.41) for neglect, and 0.26 (SD = 0.61) for household challenges. With regards to individual ACE experiences, 14% of the sample reported parental separation or divorce. Other individual ACE experiences that the sample answered affirmatively at a high rate were emotional neglect (12%), emotional and sexual abuse (both at 11%), physical abuse (6%), and mental illness in the household (5%). The percentages of students reporting physical neglect, incarcerated household member, substance abuse in the household, and mother treated violently were low, all at 3% or below. Finally, <1% of students reported that they had family members or friends who had been infected with COVID-19 (0.5%) or died of COVID-19 (0.4%). Due to low occurrence, we combined both infected and died into one category for regression analysis.

**Table 2:** Level of grit, adverse childhood experience, and COVID-19 infection.

	**Mean (S.D.)**
Grit [1-5]	3.07 (0.44)
Consistency of effort [1-5]	2.86 (0.63)
Perseverance [1-5]	3.28 (0.72)
Adverse Childhood Experience [%]	
Occurrence [No=0, Yes=1]	35.16
Three types or more	8.44
Adverse Childhood Experience [0-10]	0.69 (1.28)
Abuse [0-3]	0.28 (0.63)
Emotional abuse [0-1]	0.11 (0.31)
Physical abuse [0-1]	0.06 (0.24)
Sexual abuse [0-1]	0.11 (0.31)
Neglect [0-2]	0.15 (0.41)
Emotional neglect [0-1]	0.12 (0.33)
Physical neglect [0-1]	0.03 (0.16)
Household Challenge [0-5]	0.26 (0.61)
Parental separation or divorce [0-1]	0.14 (0.34)
Mother treated violently	0.02 (0.15)
Substance abuse in the household [0-1]	0.02 (0.14)
Mental illness in the household [0-1]	0.05 (0.21)
Incarcerated household member [0-1]	0.03 (0.16)
COVID-19 infection in family and friends [%]	
No	99.14
Infected	0.48
Dead	0.37

*N = 1,871*.

### Multivariate Analyses

Table 3 presents the standardized estimates of grit, estimated by OLS regression. Following past literature, which has measured ACEs as both dichotomous (occurrence of any ACEs at all) and continuous variables (total ACE score), we conducted two models in order to test the robustness of the association between ACEs and grit. The first modeled ACE as a dichotomous variable that measured the occurrence of any ACEs (0 = no, 1 = yes), while the second one used the observed ACE score in the analysis. When measured as a binary variable in Model 1, ACEs had a significant and negative association with grit. Students with any ACEs reported 0.09 standard deviation less grit than students without any ACEs (*p* < 0.001). Students who had family members or friends infected with COVID-19 did not have any statistically significant difference in grit scores compared to their counterparts. Overall, level of grit increased with age. The adjusted R-square of Model 1 was 0.03. The adjusted R-square kept stable at 0.03 in Model 2. Model 2 showed that the ACE score had a significant and negative association with grit. Like occurrence of ACE, a one standard deviation increase in the ACE score was associated with a 0.09 standard deviation reduction in grit (*p* < 0.001). The rest of the results of Model 2 were similar to those reported in Model 1. The small adjusted R-square values in both models suggest that a lot of variances in grit in Chinese college students were not explained in the models.

**Table 3:** Standardized estimates of grit, by OLS regression.

	**Model 1**			**Model 2**		
	**B**	**S. E**.	**P**	**B**	**S. E**.	**P**
Adverse childhood experience [No = 0, Yes = 1]	−0.09	0.02	***	–	–	
Adverse childhood experience [score]	–	–		−0.09	0.01	***
COVID-19 infection in family and friends	−0.01	0.11		0.00	0.11	
Female	0.04	0.02	+	0.04	0.02	+
Age	0.10	0.01	**	0.10	0.01	**
Household registration: city, rural before	−0.03	0.04		−0.02	0.04	
Household registration: city	0.03	0.03		0.03	0.03	
Junior	0.01	0.03		0.01	0.03	
Han	0.00	0.03		0.01	0.03	
Married	−0.02	0.03		−0.01	0.03	
Junior high school	−0.01	0.04		−0.02	0.04	
High school	0.01	0.05		−0.02	0.05	
College and above	0.02	0.05		0.00	0.05	
Family income	−0.03	0.01		−0.03	0.01	
Welfare status	0.03	0.03		0.02	0.03	
Number of family members	−0.02	0.01		−0.02	0.01	
College fixed effects	Yes			Yes		
Adjusted R-square	0.03			0.03		

*N = 1,871*.

+ *p < 0.10*,

** *p < 0.01*,

*** *p < 0.001*.

Last, we conducted robustness tests on the relationship between ACEs and grit. The same regression analyses in Table 3 were performed, but the ACE variable was replaced by a different specification: whether a student reported the occurrence of three types or more; the three ACE subscales; or individual ACE items. Each ACE specification was regressed on grit, along with other controls in Table 3. In addition to the grit scale, the analyses for the two subscales of grit, perseverance and consistency, were performed as well. Standardized results are presented in Table 4. For simplicity, we only present the standardized coefficients of ACE items in Table 4. The results for other variables were similar to those reported in Table 3. The occurrence of three or more types of ACE had a significant negative association with grit (*B* = −0.07, *p* < 0.01). Both the abuse and neglect subscales had a significant negative relation with grit. There was a greater association with abuse (*B* = −0.09, *p* < 0.001), followed by neglect (*B* = −0.06, *p* < 0.01). The household challenge subscale had no significant association with grit. Next, only three ACE items (emotional abuse, sexual abuse, and emotional neglect) were significantly associated with lower levels of grit. All had an effect size around −0.07 (*p* < 0.01). Physical abuse was marginally associated with grit, with an effect size of −0.04 (*p* < 0.10). All types of household challenges did not have any statistically significant association with grit overall.

**Table 4:** Robust tests of ACEs on grit.

	**Grit**			**Consistency**			**Perseverance**		
	**B**	**S. E**.	**P**	**B**	**S. E**.	**P**	**B**	**S. E**.	**P**
ACE Scale									
Occurrence [No = 0, Yes = 1]	−0.09	0.02	***	0.02	0.03		−0.12	0.04	***
Three types or more [No = 0, Yes = 1 for 3 ACEs or more]	−0.07	0.04	**	0.01	0.05		−0.10	0.06	***
ACE score [0–10]	−0.09	0.01	***	0.07	0.01	**	−0.16	0.01	***
Three Dimensions									
Abuse	−0.09	0.02	***	0.02	0.02		−0.13	0.03	***
Neglect	−0.06	0.02	**	0.03	0.04		−0.10	0.04	***
Household challenge	−0.04	0.02		0.10	0.03	***	−0.14	0.03	***
Individual items									
Emotional abuse [0-1]	−0.07	0.03	**	0.02	0.05		−0.11	0.05	***
Physical abuse [0-1]	−0.04	0.04	+	0.02	0.06		−0.07	0.07	**
Sexual abuse [0-1]	−0.07	0.03	**	0.01	0.05		−0.09	0.05	***
Emotional neglect [0-1]	−0.07	0.03	**	0.01	0.04		−0.09	0.05	***
Physical neglect [0-1]	−0.03	0.06		0.05	0.09		−0.08	0.10	***
Parental separation or divorce [0-1]	−0.02	0.04		0.07	0.05	*	−0.09	0.05	***
Mother treated violently	0.01	0.07		0.07	0.10	**	−0.05	0.11	*
Substance abuse in the household [0-1]	−0.04	0.08		0.07	0.11	**	−0.11	0.12	***
Mental illness in the household [0-1]	−0.03	0.05		0.05	0.06	**	−0.08	0.07	***
Incarcerated household member [0-1]	−0.03	0.06		0.03	0.09		−0.06	0.10	*

*N = 1,871*.

+ *p < 0.10*,

* *p < 0.05*,

** *p < 0.01*,

*** *p < 0.001*.

By contrast, household challenges appeared to have a significant positive association with the consistency of interests dimension of grit (*B* = 0.10, *p* < 0.01). This was reflected by the results of regressing consistency onto individual ACE items: parental separation or divorce; mother treated violently; substance abuse in the household; and mental illness in the household all showed significant positive effects on consistency of interests. Overall, each 1-point increase in ACE score was associated with a 0.07 standard deviation increase in consistency of interests (*p* < 0.01).

Interestingly, ACEs had a greater negative association with the perseverance of effort dimension. Each point increase in ACE score was associated with a 0.16 standard deviation decrease in perseverance (*p* < 0.001), as opposed to a 0.09 standard deviation decrease in grit overall (*p* < 0.001). Students who had reported experiencing at least one ACE had 0.12 standard deviation less perseverance of effort than those who did not experience any ACEs (*p* < 0.001). Those who had experienced 3 or more ACEs had 0.10 standard deviations less perseverance than those who experienced <3 (*p* < 0.001). Meanwhile, each type of ACE—abuse (*B* = −0.13, *p* < 0.001), neglect (*B* = −0.10, *p* < 0.001), and household challenges (*B* = −0.14, *p* < 0.001)—also had significant negative associations with perseverance. Finally, each individual ACE item appeared to have significant negative associations with perseverance. Emotional abuse and substance abuse in the household had the greatest negative association with perseverance (each at *B* = −0.11, *p* < 0.001), followed by sexual abuse, emotional neglect, and parental separation or divorce (each at *B* = −0.09, *p* < 0.001), physical neglect and mental illness in the household (each at *B* = −0.08, *p* < 0.01), physical abuse (*B* = −0.07, *p* < 0.01), incarcerated household member (*B* = −0.06, *p* < 0.05), and finally mother treated violently (*B* = −0.05, *p* < 0.05).

Finally, ACEs tend to have different relations with consistency. Each point increase in ACE score was associated with a 0.07 standard deviation increase in consistency (*p* < 0.01), while the occurrence of at least one ACE and the occurrence of three or more ACEs did not have any significant association with consistency. The association between ACEs and consistency came from household challenges (*B* = 0.10, *p* < 0.001), while abuse and neglect had no statistically significant relation with consistency. Specifically, parental separation or divorce (*B* = 0.07, *p* < 0.01), mother treated violently (*B* = 0.07, *p* < 0.01), substance abuse in the household (*B* = 0.07, *p* < 0.01), and mental illness in the household (*B* = 0.05, *p* < 0.05) all had significant and positive associations with consistency.

## Discussion

This study sought to examine ACEs as an antecedent of grit in Chinese college students. Guided by trauma theory (Herman, 1992), we hypothesized that ACEs would be negatively associated with grit in a sample of Chinese emerging adults. Conceptualized as traumatic experiences, ACEs may affect grit via depressive and posttraumatic stress symptoms, including dissociation and negative self-appraisal (Herman, 1992). This cannot be tested, however, without first establishing an association between ACEs and grit. Using sample data from 1,871 junior and senior students from 12 universities spread across China, we found that ACEs had negative effects on grit, though the effect size of −0.09 suggests only a small to moderate effect (*p* < 0.001). Results also indicated that not all types of ACEs nor the number of ACEs experienced were equally negatively associated with grit. Effect sizes varied when grit was regressed onto different ACE specifications. Emotional and sexual abuse, as well as emotional neglect, were most strongly negatively associated with grit overall. Meanwhile, the association between physical abuse and grit was marginally significant.

The significant negative associations between emotional and sexual abuse and emotional neglect and grit may be a result of decreased sense of relatedness. Datu (2017) found that sense of relatedness, the extent to which a person feels accepted by social partners such as parents, teachers, and friends, is positively associated with higher grit. Given that sexual and emotional victimization, as well as neglect, may lead to negative self-appraisal and feelings of rejection in a child (Rohner and Rohner, 1980; Alexander, 1992; Herman, 1992; Feiring et al., 2002), these experiences may impair attachment (Alexander, 1992) and sense of relatedness and, subsequently, grit. This is consistent with trauma theory, which has described that those children who experience severe trauma throughout childhood and adolescence experience turbulent interpersonal relationships as they grow up (Herman, 1992). Indeed, a study on motivation in Chinese adolescents have found that socioemotional relatedness to adults may be more important to sustaining adolescents' motivation than autonomy (Bao and Lam, 2008), indicating the importance of relationships in motivation and related constructs like grit in Asian contexts. This could be exacerbated for adolescents and emerging adults who live in collectivist contexts which emphasize socioemotional relatedness (Iyengar and Lepper, 1999). De Vera et al. (2015) also relayed that social support systems are an important component of grit in Asian cultures, but gaps in grit theory grounded in such contexts continues to be an area of inquiry that requires further investigation (Datu et al., 2017). Future research may use mediational studies to examine how ACEs affect the way that adolescents and emerging adults in collectivist contexts view or approach their peer and family relationships and how this, in turn, may affect grit, as there are currently limited studies on the sub-facets of grit as contextualized in non-Western cultures.

Another potential explanation for the effects of emotional and sexual abuse on grit can be formulated based on the relations between sexual abuse and mindfulness (Elices et al., 2015) and dispositional mindfulness and grit (Raphiphatthana et al., 2018). It is possible that the effects of abuse and neglect on mindfulness mediates the relationship between abuse and neglect and grit. Indeed, trauma theory (Herman, 1992) would also offer insight into these relations, as dissociation is a common posttraumatic symptom exhibited by trauma survivors, making it difficult for individuals to engage in the practice of mindfulness. Further research on this area is warranted.

Interestingly, household challenges had no significant association with students' grit, but when we regressed grit on the ACE subscales, household challenges appeared to have a significant and positive association with the consistency of interests dimension (*B* = 0.10). This finding is interesting considering that Datu et al. (2016) previously reported that consistency of interests may not be a salient construct in the measurement of grit within collectivistic cultures. Previous scholarly literature by Suh (2007) reported less behavioral consistency in collectivist cultures, which may instead encourage adaptability to situations (Datu, 2017; Datu et al., 2018a,b) to maintain relational harmony (Markus and Kitayama, 1991), however, consistency of interests may be increased by household challenges via fractured relationships and attachments (Alexander, 1992), placing individuals in more autonomous positions. An individual may be more likely to pursue their own personal interests rather than tending to the expectations of others due to a perceived lack of relatedness and acceptance at home.

When the ACE specifications were regressed on perseverance of effort dimension, all specifications—that is, more than one ACE, more than three ACEs, each type of ACE, and each individual ACE scale item—had a significant negative association with perseverance of effort. It is possible that ACEs negatively affect perseverance of effort through their deleterious effects on psychological well-being (Herman, 1992), as those with ACEs are at greater risk of developing mental illnesses such as depression (Schilling et al., 2007; Lamoureux et al., 2012; Chen et al., 2014; Merrick et al., 2017), which is associated with negative symptoms like avolition and anhedonia (Price and van Stolk-Cooke, 2015). Child sexual abuse, for example, has been linked to low self-esteem (Stern et al., 1995; Mwakanyamale and Yizhen, 2019). Those with low self-esteem may be less likely to exhibit perseverance of effort, as they may be deterred by obstacles or failure.

Our findings reflected that COVID-19 had no significant association with grit, though this result may have been caused by measurement error and low COVID-19 prevalence in China at the time of data collection. The prevalence of COVID-19 had stabilized and remained low since April 2020, when the last city, Wuhan, lifted its lockdown (Zhong and Wang, 2020). By the time that data were collected in September 2020, it had been approximately 5 months since China lifted its lockdown measures. Thus, the students were less likely to report proximity to COVID-19 infections in their lives. Further research in this area is required. We offer suggestions for future studies that seek to examine how COVID-19 has impacted the psychosocial well-being of Chinese citizens in the next section.

This study has several limitations. First, our analyses were based on a cross-sectional dataset, which can only approximate an associative relationship, rather than a causal one, among ACEs, COVID-19 infection, and grit during the pandemic. Future studies may use a longitudinal design to examine the causal relationships of these variables. Second, there were other unobserved variables, such as academic stressors and peer support, that could affect grit but were not included in the study. The absence of these unobserved variables may have effects on the estimates reported in this study. Third, the findings of this study are based on data from students of social science departments in 12 colleges across China. Although the large sample size and geographic diversity of these colleges increase our confidence, the extent to which these findings can be generalizable to all Chinese college students is unknown and requires further research. Fourth, the lack of any significant effect of COVID-19 on grit may be the result of measurement error since this was dependent on students' recall of events. There had been low prevalence of COVID-19 in China since April 2020, 5 months prior to data collection. Future research using other countries, where the COVD-19 pandemic still continues to affect daily life, is warranted. Finally, data gathered for key variables such as grit and ACEs relied on students' self-reports. Self-reporting leaves our data subject to unintended and intended reporting errors, including social desirability bias, particularly for data related to ACEs. Considering that ACEs relate to the respondent's home and filial relations, it is possible that respondents may avoid answering questions that reveal intimate details of family's life to preserve their own image as well as their family's image (Eriksson et al., 2017). Eriksson et al. (2017) found that people are willing to sacrifice their own resources to preserve another person's image when that person was considered a member of the in-group. In Chinese culture, which promotes a high-context self (Suh, 2007), saving face is especially salient when discussing matters related to the family unit. Thus, to address this concern, future studies may consider triangulating findings from different data sources, such as peer or teacher reports.

## Conclusion

This study is one of the first to examine how childhood experiences, specifically ACEs, may differentially predict grit during emerging adulthood. The results indicate that sexual abuse, emotional abuse, and neglect had significant negative associations with grit. Given that grit is particularly important to predict academic and professional success, as well as psychological well-being, those individuals who have experienced sexual and emotional victimization may experience challenges related to their education, career, and health. Our results suggest that interventions to buffer the negative effects of ACEs on grit are necessary. Mindfulness interventions have been found to improve self-esteem (Randal et al., 2015) and self-acceptance (Thompson and Waltz, 2008). Mindfulness has also been shown to positively affect resilience and mental health (Huang et al., 2019, 2020; Cheung et al., 2020), constructs that are related to grit (Musso et al., 2019; Meyer et al., 2020). Although mindfulness interventions have yet to be examined for their effects on grit, past literature has found that mindfulness and grit are significantly positively associated (Raphiphatthana et al., 2018). Our findings thus set up future studies to continue building on our knowledge of grit, including its antecedents and possible ways to bolster it in vulnerable populations, within the context of collectivistic cultures.

## Data Availability Statement

The raw data supporting the conclusions of this article will be made available by the authors, without undue reservation.

## Ethics Statement

The studies involving human participants were reviewed and approved by Review Committee, School of Public Administration, Guangdong University of Foreign Studies. Written informed consent for participation was not required for this study in accordance with the national legislation and the institutional requirements.

## Author Contributions

SC, C-CH, and CZ: conceptualization, validation, formal analysis, and writing—original draft preparation. SC and C-CH: methodology and software. C-CH and CZ: resources and investigation and data curation. All authors contributed to the article and approved the submitted version.

## Conflict of Interest

The authors declare that the research was conducted in the absence of any commercial or financial relationships that could be construed as a potential conflict of interest.
